# Dataset on electrochemical reduced graphene oxide production: Effect of synthesis parameters

**DOI:** 10.1016/j.dib.2018.10.014

**Published:** 2018-10-09

**Authors:** A.B. López-Oyama, M.A. Domínguez-Crespo, A.M. Torres-Huerta, E. Onofre-Bustamante, R. Gámez-Corrales, N. Cayetano-Castro, A.C. Ferrel-Álvarez

**Affiliations:** a Instituto Politécnico Nacional, CICATA-Altamira, Grupo CIAMS, Km 14.5, Carretera Tampico-Puerto Industrial Altamira, C.P. 89600 Altamira, Tamaulipas, Mexico; b CONACYT – CICATA-Altamira, Carretera Tampico-Puerto Industrial Altamira, C.P. 89600 Altamira, Tamaulipas, Mexico; c Universidad de Sonora, Blvd. Rodríguez y Rosales S/N, C.P. 83000 Hermosillo, Sonora, Mexico; d Instituto Politécnico Nacional, Centro de Nanociencias Micro y Nanotecnologías, C.P. 07300 Mexico, D.F, Mexico

**Keywords:** Graphite, Graphene oxide (GO), Electrochemically reduced graphene oxide (ERGO), Corrosion resistance

## Abstract

Structural and microstructural characterization combined with vibrational, rotational modes are quite important to determinate reduction degree during synthesis of reduced graphene oxide. These data and analysis support the research article “Electrochemical alternative to obtain reduced graphene oxide by pulse potential: effect of synthesis parameters and study of corrosion properties” (López-Oyama et al., 2018). The data and analysis presented here included raw data for selected reduction potentials (*V*_SCE_) and different temperatures values (°C). Transmission electron microscopy (TEM) images of the exfoliated graphite are shown to corroborate the effect of the applied voltage to obtain electrochemically reduced graphene oxide (ERGO) on commercial 304L stainless steel (304L SS). The data provided in this article has not been previously published and are available to enable critical or extended analyses.

**Specifications table**

**Table:** 

Subject area	*Materials science, nanostructures, electrochemistry*
More specific subject area	*Electrochemically reduced graphene oxide (ERGO), protective coatings, corrosion*
Type of data	*Tables, figures and text file*
How data was acquired	*Derived from a Mexican Government agreement through Laboratory Experiments. The characterization was realized by X-ray diffraction (XRD, Bruker D8 Advanced), micro Raman (XPLORA model BX41TF using argon 532 nm laser) and Transmission electron microscopy (JEM-ARM200CF, JEOL operating at 200 kV).*
Data format	*Raw, filtered, fitted curves and analyzed data.*
Experimental factors	*The data were realized on graphene powders and ERGO coated AISI 304L SS substrates*
Experimental features	*The relationship between the synthesis parameters and electrochemical performance of ERGO coated AISI 304L SS substrates was determined. Data were acquired on as-obtained samples without special treatment.*
Data source location	*TEM images were taken at the Centro de Nanociencias micro y Nanotecnologías del Instituto Politécnico Nacional C.P. 07300 México, DF, México*
	*Raman spectra were acquired at Departamento de Física, Universidad de Sonora, C.P. 83000, Sonora, México.*
	*XRD patterns were collected at Centro de investigación en Ciencia Aplicada y Tecnología Avanzada del Instituto Politécnico Nacional, C.P. 89600 Altamira, Tamaulipas, México*
Data accessibility	*All data are available with this article*
Related research article	Electrochemical alternative to obtain reduced graphene oxide by pulse potential: effect of synthesis parameters and study of corrosion properties

**Value of the data**

• This data is valuable because confirm structural changes in the reduction degree of graphene oxide in dependence of temperature.
• The data showed also a comparison of two synthesis approaches. GO production by the traditional modified Hummers’ method and thereafter applied pulse potentials (−1.6 and −2.0 *V*_SCE_) using 304L SS electrodes were used to reduce GO powders in acid solution. In the second method a potentiostatic approach varying the pulse potential from −1 to −4 *V*_SCE_ (10 min) between the 304L SS electrodes in an aqueous solution containing graphite +H_2_SO_4_, to pass from graphite→GO→ERGO.
• The data can be used to investigate optimal barrier properties of graphene in different metallic substrates.
• The data can be relevant in explore ERGO flexible films.

## 1 Data

**Fig. 1:**
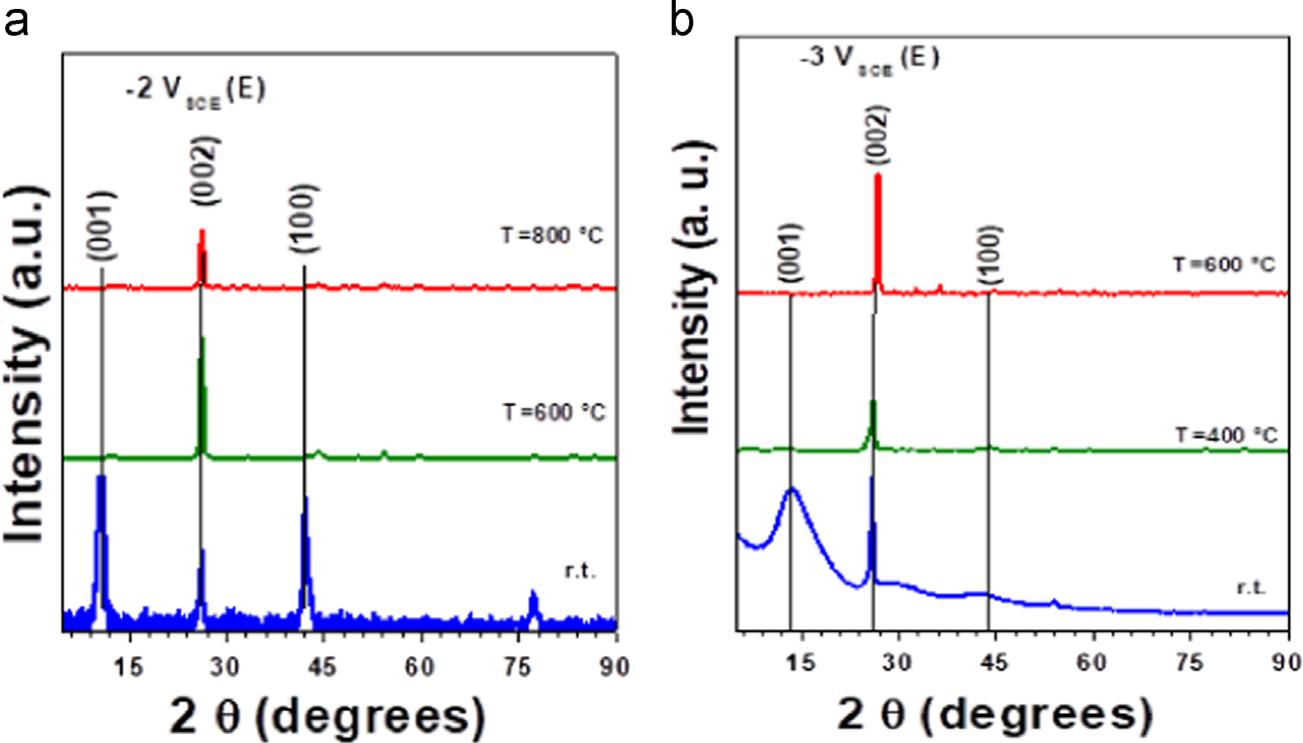
XRD patterns of selected ERGO powders obtained by one step route at −2 and −3 *V*_SCE_ and different thermal treatments (r.t., 200, 400, 600 and 800 °C).

**Fig. 2:**
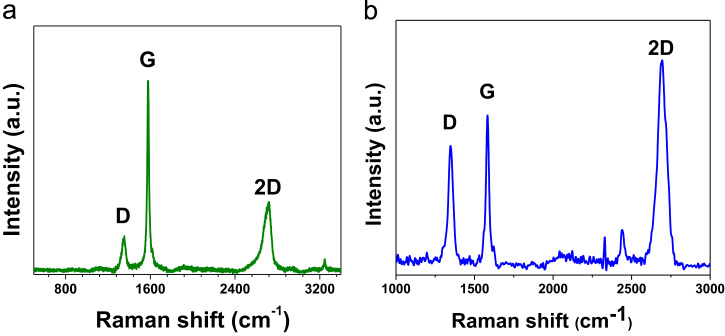
Raman spectra of (a) graphite and (b) ERGO deposited onto 304L SS electrochemically reduced at −4 *V*_SCE_. It is possible to observe that 2D feature appears more intense to G and D bands as a comparison with graphite.

**Fig. 3:**
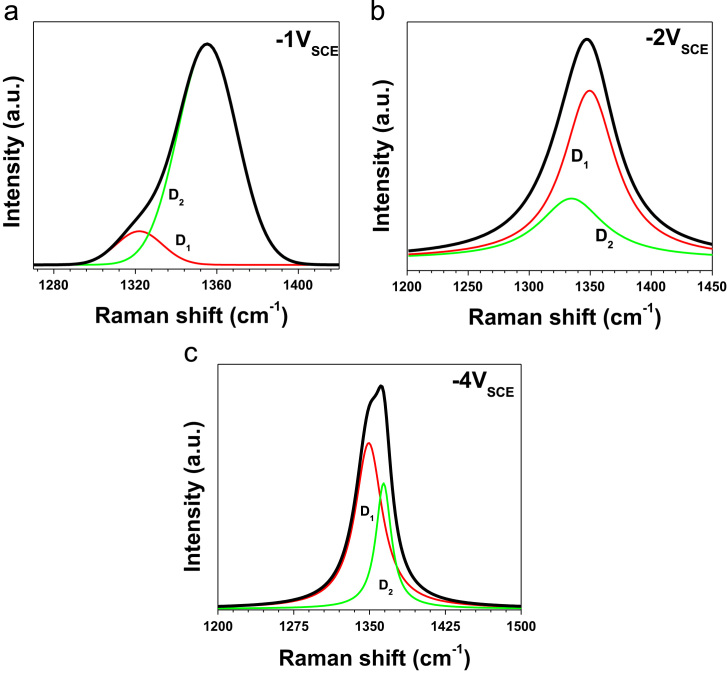
Raman spectroscopy of ERGO/304L SS samples synthesized by applying the potentiostatic approach (−1, −2 and −4 *V*_SCE_), where it is shown the fitting of D_1_ and D_2_ bands.

**Fig. 4:**
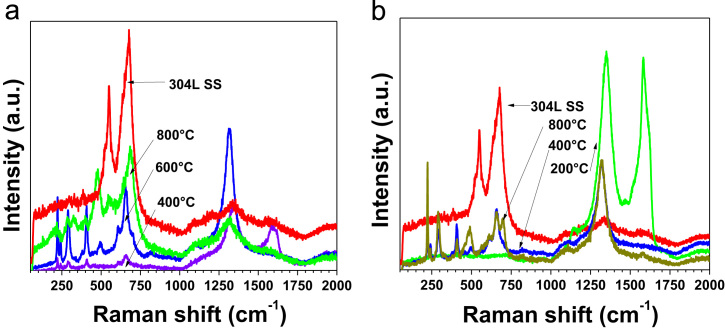
Raman features of (a) uncoated 304L SS and (b) ERGO/304L SS samples obtained at −1.6 *V*_SCE_, after being thermally treated at different temperatures. The spectra confirm a successfully ERGO deposition on the metallic substrates.

**Fig. 5:**
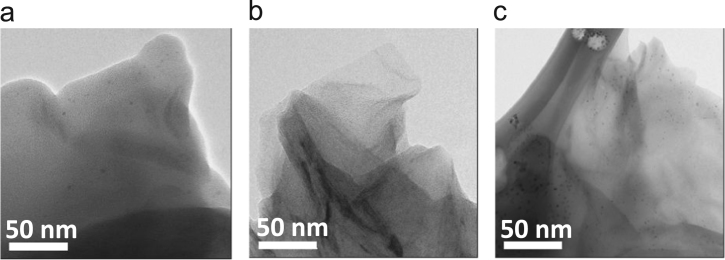
TEM images of (a) exfoliated graphite before the electrochemical reduction process, (b) ERGO at 1.6 V and (c) ERGO at 2.0 V.

The data presented in this paper are focused on structural changes and evaluation of vibrational modes in dependence of the thermal treatment. They were used as auxiliary way to enhanced reduction degree of ERGO both powders and coatings. In consequence, the data showed structural characterization with an analysis to determine a structural fingerprint during the graphite exfoliation and ERGO production (powders and coatings) via two synthesis routes on 304L stainless steel at different experimental conditions [1]. XRD data displayed the complementary information at −2 and −3 *V*_SCE_ (Fig. 1) [1]. Raman spectra were used to identify 2D bands as well as the differences between graphite and ERGO deposited on metallic substrates (Fig. 2). Raman spectra of the samples showed disorders and defects in graphene-based materials (Fig. 3)**,** but the deconvolution to fit D_1_ and D_2_ bands was only possible when pulse electrodeposition technique was used for ERGO film production, i.e. combining chemical–electrochemical methods produce samples with a high fluorescence. The data of Fig. 4 shows Raman features that correspond to 304L SS uncoated and thermally treated samples after ERGO deposition on metallic substrates. The G and 2D bands of Raman shift evidenced successfully ERGO deposition. Table 1 displays the obtained data from Raman fitting on coated 304L SS substrates. The characterization showed that the quantity of defects and desirable features could be modulate with the synthesis parameters, which is promissory for future applications [2], [3], [4], [5]. Transmission electron microscopy (TEM) images also confirm microstructural differences between exfoliated graphite and powders electrochemically reduced (Fig. 5)**.**

**Table 1:** Data obtained from Raman fitting on coated 304L SS substrates where it is seen FWHM, area, peak position, *I*_D_/*I*_G_ and *I*_2D_/*I*_G_ ratio.

***V***_**appl**_**(*V***_**SCE**_**)**		**G-Band**			**D-Band**			**2D-Band**			**Ratio**		
		**FWHM**	**Area (cm**^**2**^**)**	**Peak position (cm**^**−1**^**)**	**FWHM**	**Area (cm**^**2**^**)**	**Peak position (cm**^**−1**^**)**	**FWHM**	**Area (cm**^**2**^**)**	**Peak position (cm**^**−1**^**)**		***I***_**D**_**/*I***_**G**_	**1**_**2D**_**/*I***_**G**_
One-step route	−1	20.76	10,391.95	1582.9	41.48	2266.59	1332.43	72.35		2711		0.27	0.33
	−2	24.69	9284.77	1581.85	63.30	3727.62	1353.88	74.01		2709		0.28	0.40
	−3	–	–	–	–	–	–	–	–			–	–
	−4	19.20	3163.80	1583.2	37.17	1350.48	1356.13	71.58	–	2693		0.2	0.41–1.35
Two-step route	−1.6	–	–	–	–	–	–	–	–	–	–	–	–
	−2.0	–	–	–	–	–	–	–	–	–		–	–

## 2 Experimental design, materials and methods

### 2.1 Synthesis

ERGO powders and films were synthesized by two methods onto 304L stainless steel plates (30 × 30 × 2 mm). *First method.* GO was produced from graphite powders using a modification of the Hummers´ method [6]; then, ERGO powders and films were obtained from a stirred acid solution (H_2_SO_4_,(30% (v/v)) containing GO powders. Two-step potentials of −1.6 and −2 *V*_SCE_ were evaluated during 10 min. The ERGO was filtered, washed and dried at 80 °C for 12 h, when changes in the solution color and a film on the electrode was observed [6]. Thereafter, ERGO samples were thermally treated to improve the reduction degree in air at 600, 700, 800 or 900 °C using a heating rate of 4 °C min^−1^. *Second method.* 5 g of graphite were dissolved in 30% H_2_SO_4_ (v/v), stirred, sonicated and stabilized (1 h). Thereafter, ERGO films or powders were obtained by electrochemical exfoliation using an arrangement that consisted of two 304L SS electrodes and a BK precision 1746B DC power supply. Different step-potentials from −1 to −4 *V*_SCE_ were applied during 10 min at pH 1–2. The supernatant was dried in an oven at 80 °C for 5 days.

### 2.2 Characterization

Powders and coated specimens were characterized by XRD (Bruker D8 Advanced) Lynxeye detector, with CuKα radiation (*λ* = 0.15406 nm). The data were collected between scattering angles (2*θ*) of 5° and 90° and at a scan rate of 2° min^−1^. The crystallite size was calculated from Scherrer equation [7]. Raman spectra were acquired in a micro-Raman XPLORA model BX41TF using an argon 532 nm laser line as excitation source, with grating of 1800 and 2400 grooves mm^−1^ and a power of 250 mW, with acquisition time of 10 s. The microstructure of the as-synthesized ERGO powders were investigated by means of a JEM-ARM200CF, JEOL electron microscope, operating at 200 kV.
